# MicroRNA-26a prevents endothelial cell apoptosis by directly targeting TRPC6 in the setting of atherosclerosis

**DOI:** 10.1038/srep09401

**Published:** 2015-03-24

**Authors:** Yong Zhang, Wei Qin, Longyin Zhang, Xianxian Wu, Ning Du, Yingying Hu, Xiaoguang Li, Nannan Shen, Dan Xiao, Haiying Zhang, Zhange Li, Yue Zhang, Huan Yang, Feng Gao, Zhimin Du, Chaoqian Xu, Baofeng Yang

**Affiliations:** 1 Department of Pharmacology (State-Province Key Laboratories of Biomedicine- Pharmaceutics of China, Key Laboratory of Cardiovascular Research, Ministry of Education), Harbin Medical University, Harbin 150081, China; 2 Institute of Cardiovascular Research, Harbin Medical University, Harbin 150081, China; 3 Institute of Clinical Pharmacy, The Second Affiliated Hospital of Harbin Medical University, Harbin 150081, China

## Abstract

Atherosclerosis, a chronic inflammatory disease, is the major cause of life-threatening complications such as myocardial infarction and stroke. Endothelial apoptosis plays a vital role in the initiation and progression of atherosclerotic lesions. Although a subset of microRNAs (miRs) have been identified as critical regulators of atherosclerosis, studies on their participation in endothelial apoptosis in atherosclerosis have been limited. In our study, we found that miR-26a expression was substantially reduced in the aortic intima of ApoE^−/−^ mice fed with a high-fat diet (HFD). Treatment of human aortic endothelial cells (HAECs) with oxidized low-density lipoprotein (ox-LDL) suppressed miR-26a expression. Forced expression of miR-26a inhibited endothelial apoptosis as evidenced by MTT assay and TUNEL staining results. Further analysis identified TRPC6 as a target of miR-26a, and TRPC6 overexpression abolished the anti-apoptotic effect of miR-26a. Moreover, the cytosolic calcium and the mitochondrial apoptotic pathway were found to mediate the beneficial effects of miR-26a on endothelial apoptosis. Taken together, our study reveals a novel role of miR-26a in endothelial apoptosis and indicates a therapeutic potential of miR-26a for atherosclerosis associated with apoptotic cell death.

Atherosclerosis is the leading cause of death and disability worldwide^1^. Endothelial cell (EC) apoptosis is a crucial process for the development of atherosclerosis^2^,^3^. The endothelium may lose the ability to regulate lipid homeostasis, immunity and inflammation because of endothelial cell apoptosis^4^. Endothelial cell injury can break the integrity and the barrier function of endothelium, and facilitate the deposition of lipids, leading to atherogenesis^5^. In addition, endothelial cell apoptosis is responsible for plaque instability because endothelial cell death can predispose to arterial thrombosis^6^, causing acute coronary occlusion and sudden death. However, the mechanisms underlying endothelial cell apoptosis remain poorly understood.

MicroRNAs (miRs) are endogenous, ~22-nucleotide noncoding RNAs that negatively regulate human genes and play important roles in pathophysiological processes^7^,^8^. Accumulating evidence has implicated miRNAs as essential regulators of atherosclerosis by targeting important factors or key pathways^9^. miR-21 can dictate vascular smooth muscle cell (VSMC) fate by inhibiting apoptosis and promoting proliferation^10^. Smooth muscle cell-specific overexpression of miR-145 markedly reduces atherosclerotic plaques in ApoE^−/−^ mice^1^. Systemic delivery of miR-181b attenuates atherosclerosis by targeting NF-κB signaling in endothelial cells^11^. However, whether miRNAs also participate in regulating endothelial cell apoptosis remains largely unexplored.

miR-26a is a highly conserved miRNA that plays essential roles in development, cell differentiation and growth. It is frequently dysregulated in cardiovascular diseases such as cardiac hypertrophy^12^,^13^, atrial fibrillation^14^ and myocardial ischemia^15^. Microarray analysis revealed that the miR-26 level is reduced by 65% in aortic valve samples of patients with aortic stenosis (AS)^16^. The present study was designed to investigate the role of miR-26a in endothelial cell apoptosis in the setting of atherosclerosis and the underlying mechanisms.

## Results

### Expression of miR-26a in the aortic intima of ApoE^−/−^ mice

**Figure 1:**
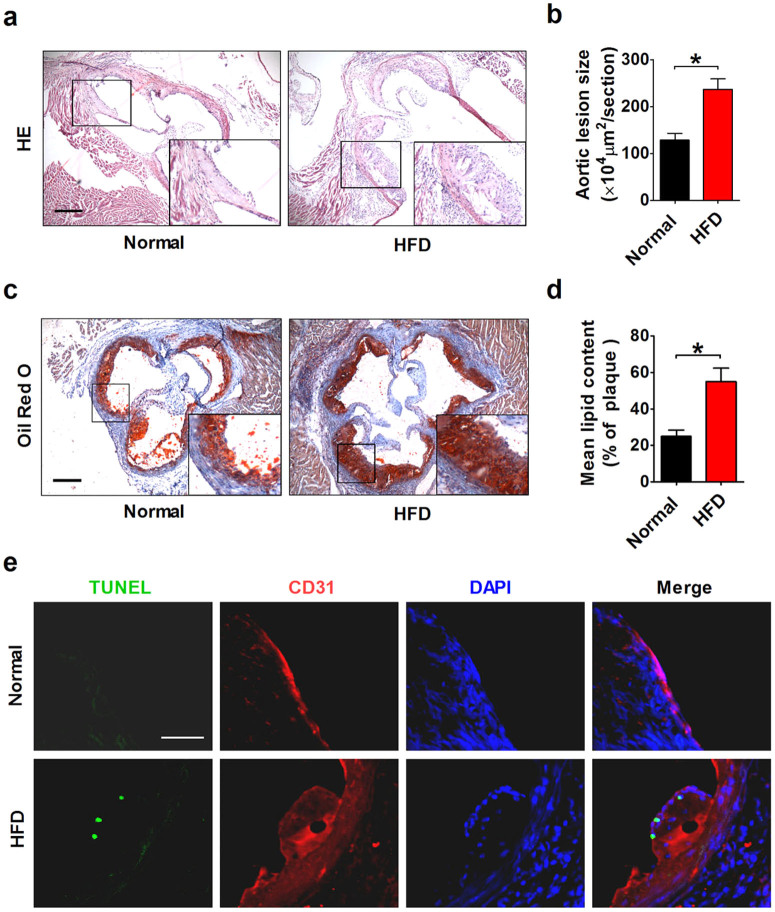
Characterization of atherosclerotic lesions in ApoE^−/−^ mice. (a) HE staining of aortic root sections showing the atherosclerotic lesions in ApoE^−/−^ mice treated with or without a 12-week high-fat diet (HFD). Scale bar indicates 600 μm. (b) Quantification of the lesion area per section in the normal-diet and HFD groups. n = 3–4 mice in each group. (c) Oil red O staining of aortic root sections showing the lipid deposition in atherosclerotic lesions. Scale bar indicates 600 μm. (d) Quantification of lipid content. n = 3–4 mice in each group. (e) Representative images of TUNEL staining showing apoptotic cells (stained in green) in the endothelium. The nuclei were stained blue with DAPI. CD31 (stained in red) was used as an endothelial marker. Scale bar indicates 50 μm. The data are presented as the mean ± S.E.M., *p < 0.05.

**Figure 2:**
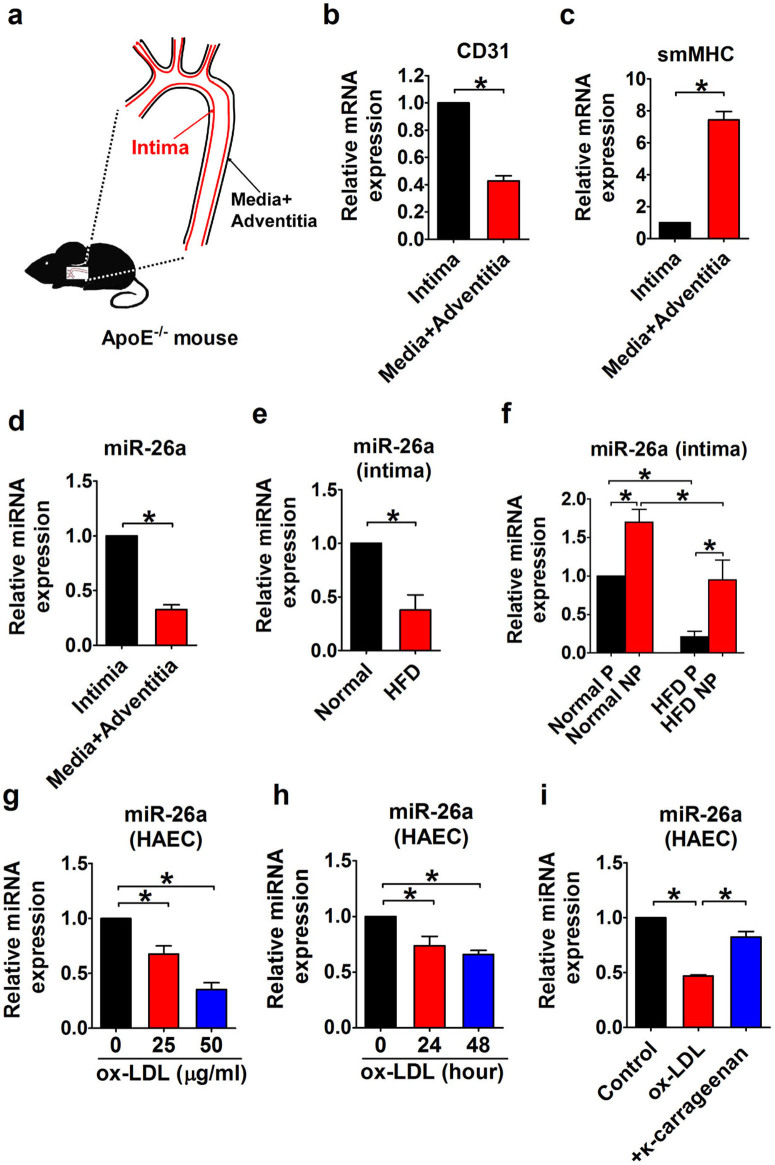
Downregulation of miR-26a in aortic intima of HFD-treated ApoE^−/−^ mice and ox-LDL-treated HAECs. (a) Preparation of aortic intima samples for PCR analysis. (b and c) Real-time RT-PCR analysis of the endothelial cell marker CD31 and the smooth muscle cell marker smMHC in the aortic intima, media and adventitia. n = 4 mice. (d) Real-time RT-PCR analysis of miR-26a in aortic intima, media and adventitia. n = 18 mice. (e) miR-26a expression in aortic intima. n = 4 mice in each group. (f) miR-26a levels in predilection (P) and nonpredilection (NP) sites of the aortic intima. n = 5 mice in each group. (g) miR-26a expression in HAECs treated with ox-LDL at different concentrations. n = 4 batches of cells. (h) miR-26a expression in HAECs treated with ox-LDL (25 μg/ml) for 0, 24, or 48 hours. n = 5 batches of cells. (i) miR-26a levels in the presence of LOX1 antagonist κ-carrageenan (125 μg/ml, 24 h). n = 5 batches of cells. The data are presented as the mean ± S.E.M., *p < 0.05.

miR-26a expression in aortic intima was first examined under condition of significant endothelial cell apoptosis. After a 12-week high-fat diet (HFD) treatment, atherosclerotic lesions were notably increased in ApoE^−/−^ mice. Analysis of histological sections of the aortic sinus stained with HE or Oil red O revealed an 84% increase in lesion area and a 120% increase in lipid content, suggesting that HFD successfully induced a severe atherosclerosis (Figure 1a–1d). TUNEL staining, along with immunofluorescent staining of CD31 (red), an endothelial cell marker, was performed to assess endothelial apoptosis in the aorta of ApoE^−/−^ mice. As shown in Figure 1e, TUNEL staining revealed that apoptosis of endothelial cells was substantially enhanced by HFD, whereas apoptosis was barely detectable in the normal-diet group, indicating that HFD induced endothelial apoptosis in ApoE^−/−^ mice. To examine whether miR-26a expression was altered during endothelial apoptosis, aortic intima was harvested for real-time RT-PCR analysis (Figure 2a). The result showed that mRNA expression of the endothelial cell marker CD31 was robustly enriched in the intima compared with media plus adventitia (Figure 2b). Conversely, expression of smooth muscle cell marker smMHC was barely detectable in the intima (Figure 2c), indicating that the isolated aortic intima contains high purity ECs. Notably, compared with the intima, miR-26a expression was lower in the media plus adventitia, suggesting that miR-26a is more abundant in ECs (Figure 2d). This result was further confirmed by in situ hybridization for miR-26a in the aorta (see Supplementary Fig. S1 online). As shown in Figure 2e, miR-26a expression was significantly reduced in the aortic intima of ApoE^−/−^ mice on HFD (p < 0.05). To further investigate whether miR-26a expression differed between the predilection and nonpredilection sites of atherosclerosis, the intima of aortic arch (predilection sites) and the thoracoabdominal aorta (nonpredilection sites) was harvested. As shown in Figure 2f, compared with nonpredilection sites, miR-26a expression was lower at the predilection sites of ApoE^−/−^ mice in the groups with or without HFD, suggesting that disturbed laminar flow influences miR-26a expression. Furthermore, we demonstrated that miR-26a was downregulated by the low shear stress generated by partial ligation of the carotid artery in ApoE^−/−^ mice^17^ (see Supplementary Fig. S2 online). Because HFD, disturbed laminar flow and low shear stress that are known to promote lesion development in atherosclerosis all were able to induce EC apoptosis, it is conceivable that miR-26a might be involved in endothelial cell apoptosis in atherosclerosis.

### miR-26a downregulation in ox-LDL-treated endothelial cells

Next, we treated human aortic endothelial cells (HAECs) with 0, 25, and 50 μg/ml oxidized low-density lipoprotein (ox-LDL), a well-known atherogenic factor that can induce endothelial cell apoptosis, for 24 h. miR-26a expression was suppressed by ox-LDL in a dose-dependent manner (Figure 2g), and the suppression was enhanced with time (Figure 2h). Lectin-like oxidized low-density lipoprotein receptor-1 (LOX1) is the main ox-LDL receptor of endothelial cells, and ox-LDL, through LOX1, contributes to the induction of endothelial dysfunction, including endothelial apoptosis^18^. To investigate whether LOX1 mediates the effect of ox-LDL on miR-26a expression, we used the LOX1 antagonist κ-carrageenan^19^. The results clearly indicated that in the presence of κ-carrageenan (125 μg/ml, 24 h), ox-LDL-induced miR-26a inhibition was rescued (Figure 2i), indicating that LOX1 mediates the effect of ox-LDL on miR-26a expression.

### miR-26a overexpression inhibits endothelial cell apoptosis

**Figure 3:**
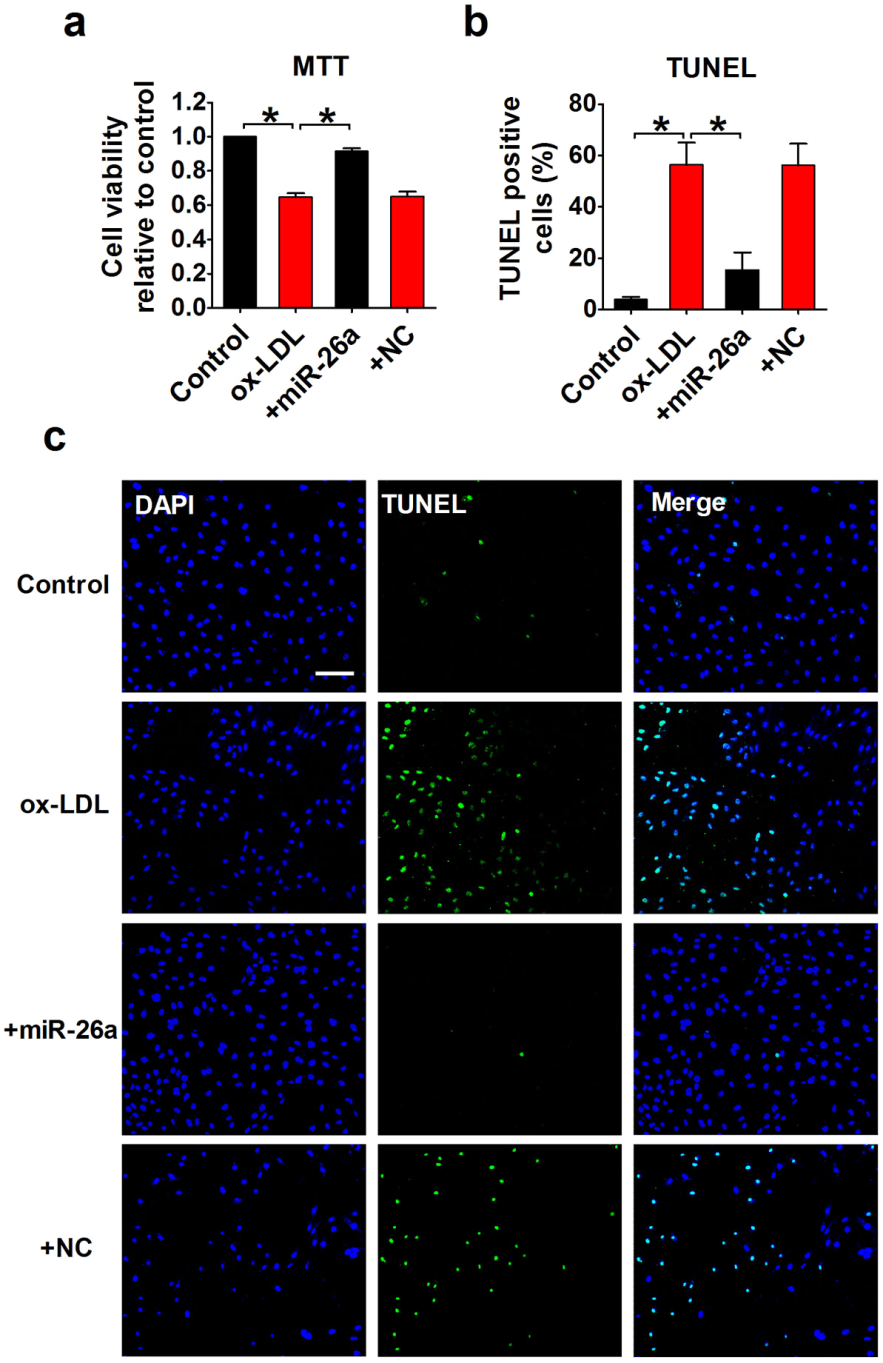
Inhibition of ox-LDL-induced apoptosis by miR-26a overexpression in HAECs. +miR-26a indicates the co-application of miR-26a mimics and ox-LDL (50 μg/ml). +NC indicates the co-application of the miRNA negative control and ox-LDL (50 μg/ml). (a) HAEC viability was detected using MTT assay. n = 6 batches of cells. (b) Percentage of TUNEL-positive cells. n = 5 batches of cells. (c) Representative images of TUNEL staining showing apoptotic cells (stained in green). The nuclei were stained blue with DAPI. Scale bar indicates 100 μm. The data are presented as the mean ± S.E.M., *p < 0.05.

The effect of miR-26a on endothelial cell survival was evaluated using MTT assay. Treatment of HAECs with 50 μg/ml ox-LDL resulted in a significant decrease in cell viability (p < 0.05). Overexpression of miR-26a inhibited the ox-LDL-induced decrease of cell viability, whereas treatment with the miRNA negative control construct had no effect (Figure 3a). To verify that the changes in cell viability observed in our experiments were at least partially attributable to apoptotic cell death, we performed TUNEL assay to detect DNA fragmentation. The percentage of cells with positive TUNEL staining was markedly increased in the ox-LDL group compared with the control group (56.32 ± 8.69% for ox-LDL *vs.* 3.88 ± 1.01% for control, p < 0.05). Transfection with miR-26a was sufficient to abolish ox-LDL-induced apoptosis, and the TUNEL-positive cells decreased to 15.50 ± 6.74% (p < 0.05 *vs.* ox-LDL; Figure 3b and c).

### Validation of TRPC6 as a target for miR-26a

**Figure 4:**
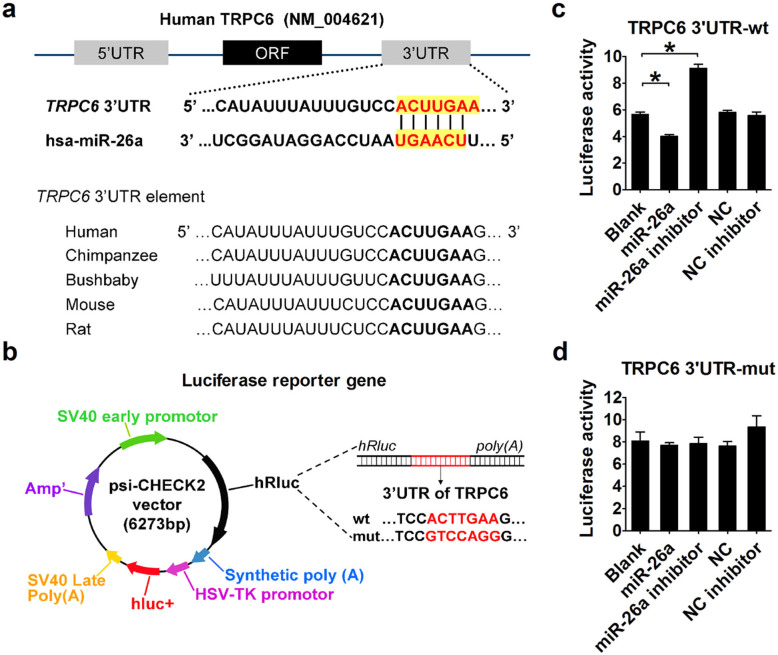
Experimental validation of TRPC6 as a target gene of miR-26a. (a) Upper panel, sequence complementarity between miR-26a and human TRPC6 3'-UTR. The letters in red indicate matched bases. Lower panel, sequence conservation of the miR-26a binding site among the human, chimpanzee, bushbaby, mouse, and rat. (b) Luciferase reporter constructs containing 3'UTR of TRPC6 or mutated 3'UTR of TRPC6. (c and d) Luciferase activities with wild-type (wt) or mutant (mut) 3'UTR of TRPC6. n = 3 batches of cells. The data are presented as the mean ± S.E.M., *p < 0.05.

To understand the mechanisms by which miR-26a inhibits endothelial apoptosis, we applied several miRNA target prediction algorithms (including DIANAmT, miRanda, miRWalk, PICTAR5, and TargetScan) to identify the potential target genes of miR-26a. All databases predict transient receptor potential canonical 6 (TRPC6) as a potential target for miR-26a. Intriguingly, TRPC6 expression was found to be up-regulated during podocyte apoptosis^20^. More importantly, silencing TRPC6 inhibits AngII-induced apoptosis in podocytes^21^. The TRPC6-encoding mRNA contains a 3'UTR binding site for miR-26a, and the complementary sequence is evolutionarily conserved among the human, chimpanzee, bushbaby, mouse, and rat mRNA orthologs (Figure 4a). Luciferase reporters containing the 3'UTR of TRPC6 or a mutated 3'UTR of TRPC6 in the miR-26a binding site were constructed and transfected into 293T cells (Figure 4b). We found that miR-26a markedly inhibited the luciferase activity of the vector containing the wild-type binding site, whereas the miR-26a inhibitor increased luciferase activity (Figure 4c). Moreover, transfection of miR-26a failed to affect the luciferase activity of the reporter carrying the mutated miR-26a binding site (Figure 4d).

### TRPC6 expression is repressed by miR-26a

**Figure 5:**
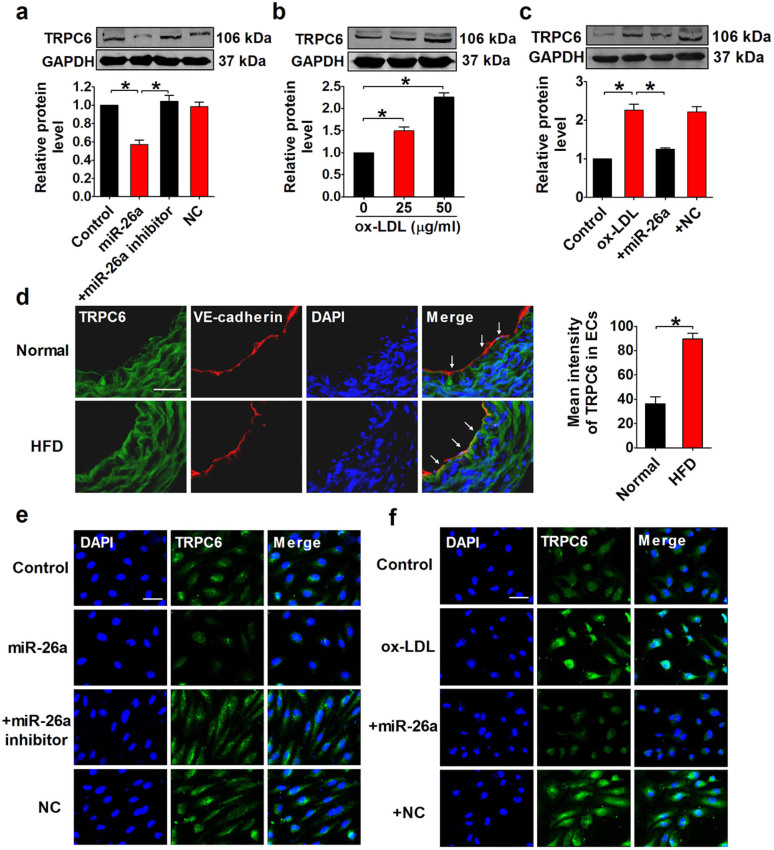
Repression of TRPC6 expression by miR-26a in HAECs. (a) Effect of miR-26a on the protein level of TRPC6. +miR-26a inhibitor indicates the co-application of miR-26a mimics and miR-26a inhibitor. NC indicates miRNA negative control. n = 5 batches of cells. Cropped blots are shown. Full-length blots are presented in Supplementary Fig. S3 online. (b) Effect of ox-LDL on the protein level of TRPC6. n = 5 batches of cells. Cropped blots are shown. Full-length blots are presented in Supplementary Fig. S4 online. (c) The ability of miR-26a to repress ox-LDL-induced TRPC6 expression. +miR-26a indicates the co-application of miR-26a mimics and ox-LDL (50 μg/ml). +NC indicates the co-application of miRNA negative control and ox-LDL (50 μg/ml). n = 5 batches of cells. Cropped blots are shown. Full-length blots are presented in Supplementary Fig. S5 online. (d) Frozen sections of aortic root were stained for TRPC6 (green) and VE-cadherin (red). The nuclei were stained blue with DAPI. Scale bar indicates 50 μm. Arrows indicate differential TRPC6 expression. TRPC6 expression was quantified in vascular ECs. n = 5 mice in each group. (e and f) Representative images of immunofluorescence staining for TRPC6 in HAECs. The nuclei were stained blue with DAPI. Scale bar indicates 50 μm. The data are presented as the mean ± S.E.M., *p < 0.05.

To confirm the regulatory effect of miR-26a on TRPC6 in HAECs, we examined the changes in TRPC6 protein level following miR-26a transfection. Western blot (Figure 5a) and immunofluorescence (Figure 5e) analyses indicated that miR-26a significantly suppressed TRPC6 expression (p < 0.05), which was reversed by co-application of miR-26a antisense inhibitor. Furthermore, ox-LDL treatment dose-dependently increased TRPC6 expression (Figure 5b), which was inversely correlated with miR-26a level (Figure 2g). Moreover, ApoE^−/−^ mice that received HFD exhibited a 53% increase in TRPC6 expression in the endothelium compared with normal diet-treated ApoE^−/−^ mice (Figure 5d). Overexpression of miR-26a inhibited the ox-LDL-induced TRPC6 upregulation (Figure 5c). A similar result was obtained from an immunofluorescence assay (Figure 5f). These findings indicate that TRPC6 acts as a downstream effector of miR-26a in HAECs.

### miR-26a inhibits endothelial apoptosis through targeting TRPC6

**Figure 6:**
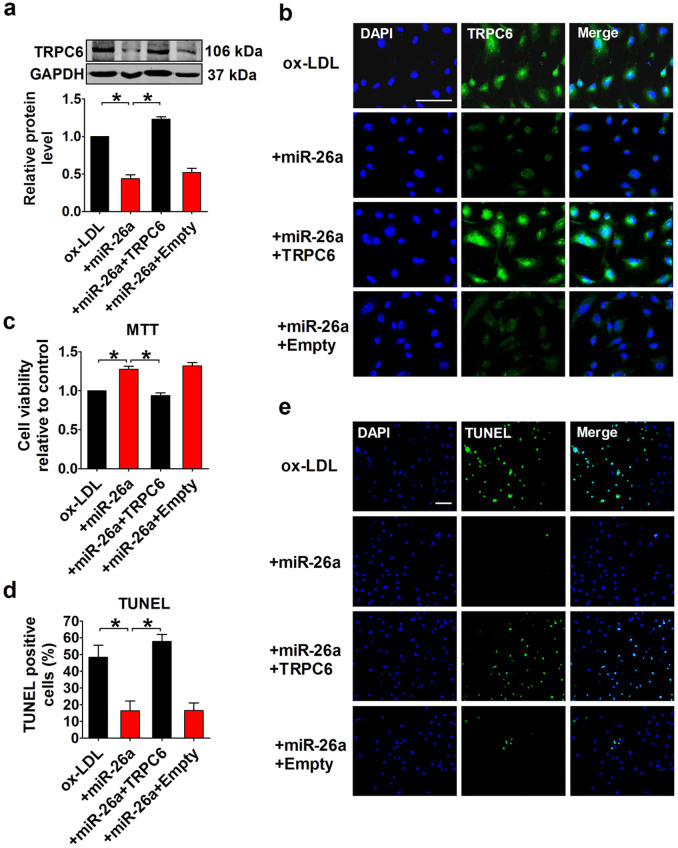
TRPC6 overexpression inhibits the anti-apoptotic effect of miR-26a in HAECs. (a) Protein levels of TRPC6 detected by Western blot. +miR-26a indicates co-application of miR-26a mimics and ox-LDL (50 μg/ml). +miR-26a+TRPC6 indicates the co-application of miR-26a mimics, ox-LDL (50 μg/ml) and TRPC6 plasmids. +miR-26a+Empty indicates the co-application of miR-26a mimics, ox-LDL (50 μg/ml) and empty plasmids. n = 5 batches of cells. Cropped blots are shown. Full-length blots are presented in Supplementary Fig. S6 online. (b) Representative images of immunofluorescence staining for TRPC6 in HAECs. The nuclei were stained in blue with DAPI. Scale bar indicates 100 μm. (c) MTT assay. n = 4 batches of cells. (d and e) TUNEL staining and the averaged data of apoptotic (TUNEL-positive) cell ratio. Scale bar indicates 100 μm. n = 5 batches of cells. The data are presented as the mean ± S.E.M., *p < 0.05.

To validate the role of TRPC6 in mediating the anti-apoptotic action of miR-26a in HAECs, we performed a rescue experiment. As shown in Figure 6a and b, miR-26a inhibited TRPC6 expression, whereas co-transfection with a TRPC6-expressing plasmid significantly increased TRPC6 protein levels (p < 0.05), as indicated by both western blot analysis and immunofluorescence staining. MTT analysis demonstrated that miR-26a inhibited ox-LDL-induced apoptosis, but forced expression of TRPC6 abolished the beneficial effect of miR-26a (Figure 6c). TUNEL staining further confirmed that TRPC6 overexpression limited the ability of miR-26a to suppress apoptosis as indicated by the increase of TUNEL-positive cells with TRPC6 overexpression (57.95 ± 4.09% for miR-26a+TRPC6 *vs.* 16.38 ± 5.90% for miR-26a, p < 0.05; Figure 6d and e).

### Overexpression of miR-26a inhibits cytosolic calcium overload and the mitochondrial apoptotic pathway

**Figure 7:**
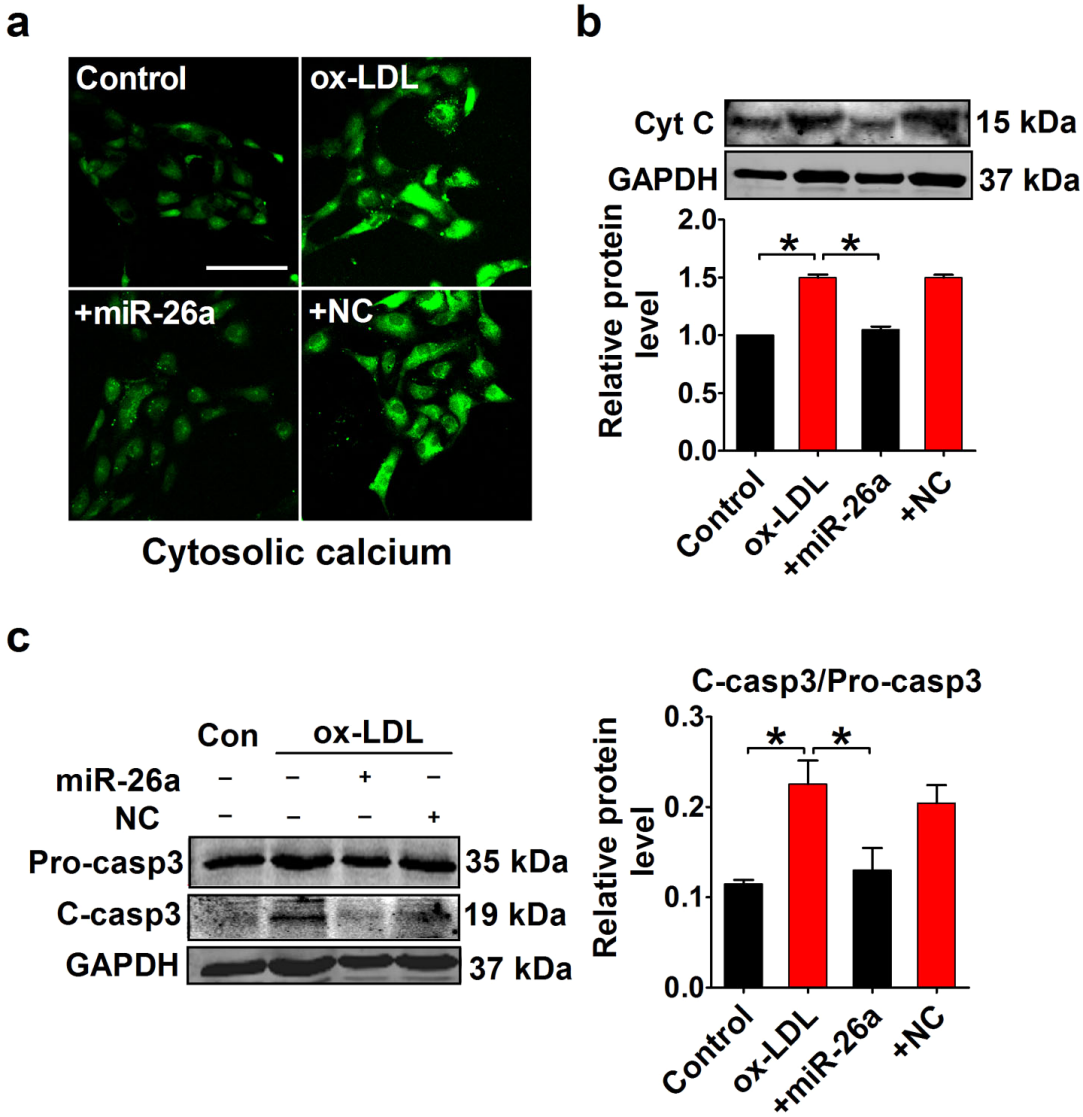
miR-26a prevents cytosolic calcium overload and subsequent apoptotic events. (a) Confocal laser scanning microscopic examination of HAECs loaded with Fluo-3/AM showing cytosolic calcium. Scale bar indicates 100 μm. (b) Protein levels of cytochrome c (Cyt C) detected by Western blotting. n = 5 batches of cells. Cropped blots are shown. Full-length blots are presented in Supplementary Fig. S7 online. (c) Western blot analysis of proform and active cleaved form of caspase3. Pro-casp3 indicates proform of caspase-3 and C-casp3 indicates the cleaved form of caspase-3. n = 5 batches of cells. Cropped blots are shown. Full-length blots are presented in Supplementary Fig. S8 online. The data are presented as the mean ± S.E.M., *p < 0.05.

TRPC6 is a calcium-permeable channel subunit, and excessive activation of TRPC6 can increase intracellular calcium. Because calcium is a key activator of the mitochondrial apoptotic pathway, we explored whether miR-26a inhibited apoptosis through the calcium-activated apoptotic pathway. Changes of cytosolic calcium were visualized by confocal laser scanning microscopy. Calcium signal was increased in HAECs treated with ox-LDL, whereas fluorescence intensity was decreased with transfection of miR-26a (Figure 7a). The apoptotic cascade was activated following the calcium overload triggered by ox-LDL, as evidenced by the release of cytochrome c and activation of caspase-3 (increased protein level of cleaved caspase-3). By contrast, the elevated cytochrome c and cleaved caspase-3 levels were decreased to the baseline level when miR-26a was overexpressed (Figure 7b and c). These data suggest that miR-26a inhibits ox-LDL-induced apoptosis through regulating intracellular calcium and the subsequent apoptotic events.

## Discussion

**Figure 8:**
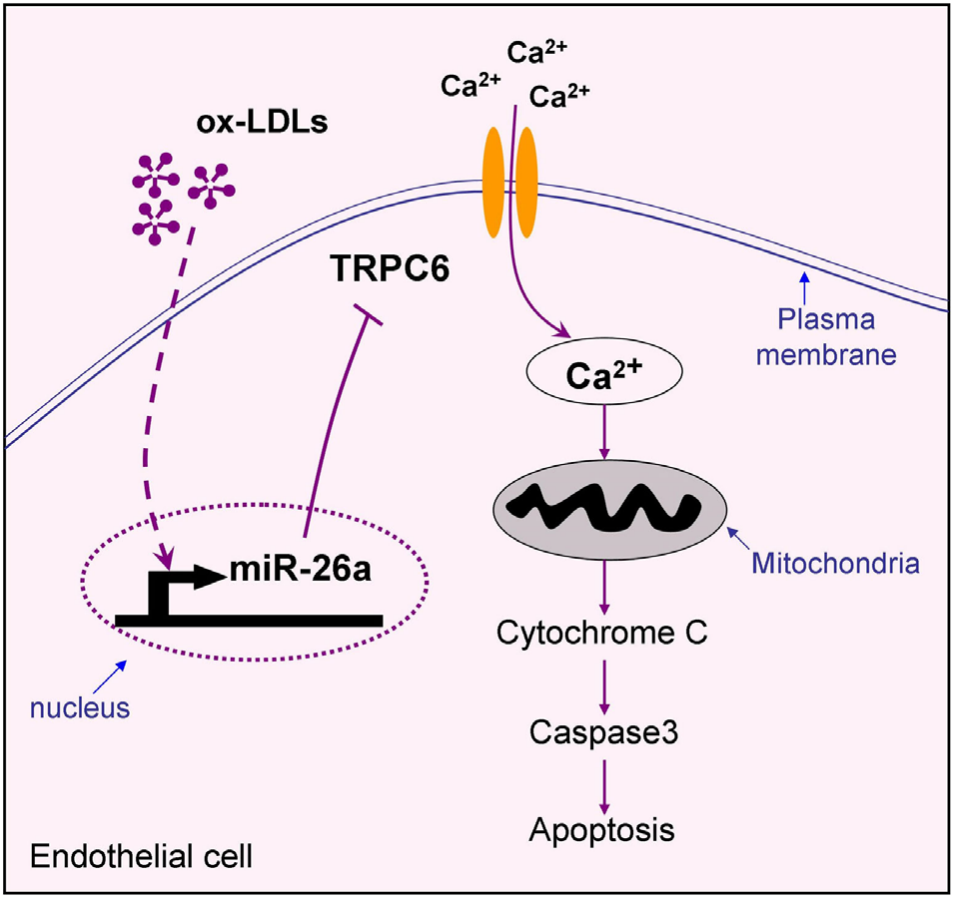
Schematic diagram of endothelial cell apoptotic signaling and protective effect of miR-26a. Ox-LDL triggers a complex cell death signaling including suppression of miR-26a and upregulation of TRPC6, leading to massive calcium influx, cytochrome c release from mitochondrial and caspase-3 activation. The data reported in this study demonstrate that miR-26a inhibits endothelial apoptosis via suppressing TRPC6 expression, thereby preventing calcium influx and subsequent activation of the mitochondrial apoptotic pathway.

The present study demonstrated miR-26a as an essential mediator for endothelial apoptosis both in vivo and in vitro. In HAECs, miR-26a overexpression was sufficient to reverse ox-LDL-induced apoptosis. The underlying mechanisms likely involve repression of TRPC6 and the associated downstream apoptotic pathway, as summarized schematically in Figure 8. This study provides evidence that miR-26a may be a novel therapeutic target for vascular diseases, such as atherosclerosis.

Atherosclerosis is a complex immunoinflammatory disease of medium and large-sized arteries^22^. Endothelial cells^4^, macrophages^23^, and smooth muscle cells^24^ are the critical players in the development of atherosclerosis. Importantly, injury of the vascular endothelium may be an initial step in the pathogenesis of atherosclerosis. Pro-atherosclerotic factors such as high glucose^25^, angiotensin II^26^ and reactive species^27^ are all able to induce apoptosis of endothelial cells. Ox-LDL, which plays a crucial role in lesion formation, stimulates endothelial cell suicide death program through several caspase-dependent or -independent pathways^28^.

Aberrantly expressed miRNAs often participate in the regulation of endothelial cell survival/death. A recent report showed that miR-126-5p affects the proliferative reserve of ECs and thereby impairs endothelial regeneration^17^. Moreover, endothelial apoptosis leads to the release of miR-126 in apoptotic bodies, which reduces plaques^29^. In our study, we found that miR-26a expression was downregulated in the aortic intima of ApoE^−/−^ mice fed with HFD and the apoptotic HAECs treated with ox-LDL, indicating the regulation of endothelial apoptosis by miR-26a. In addition, miR-26a overexpression suppressed ox-LDL-induced apoptosis in HAECs, suggesting that, in addition to the established miRNAs (Let-7g^30^, miR-29b^31^, miR-21^32^,^33^, miR-223^34^), miR-26a is another key player in regulating cell apoptosis. In fact, miR-26 has been consistently reported to play diverse roles in cardiovascular diseases. Zhang et al. demonstrated that miR-26 expression is reduced in cardiac hypertrophy induced by transverse abdominal aortic constriction (TAAC) surgery^12^. Overexpression of miR-26a in a mouse model of atrial fibrillation (AF) markedly reduced AF incidence induced by intracardiac pacing^14^. In addition, a recent study indicated that miR-26a serves as an anti-angiogenic factor and reduction of miR-26a can promote angiogenesis in endothelial cells^35^. Our study identified a new role for miR-26a in endothelial cells, the anti-apoptotic effect.

The mechanisms underlying the regulation of endothelial apoptosis by miRNA are often attributed to targeting important factors or the key pathways related to apoptosis. Let-7g was reported to negatively regulate endothelial cell apoptosis by targeting caspase-3 expression^30^. miR-29b augments endothelial permeability and apoptosis through inhibition of MT1 expression and thus regulates caspase-3 activity^31^. miR-21 protects against high glucose-induced endothelial cytotoxicity by targeting death-domain associated protein^33^. In this study, we identified TRPC6 as a direct target of miR-26a with its repression mediating the anti-apoptotic action of miR-26a. TRPC6, belonging to a large family of TRPC channels, is a calcium-permeable channel expressed in a variety of cell types, including ECs^36^. The relationship between TRPC6 and apoptosis has been well investigated in podocytes^21^,^37^,^38^,^39^. For example, TRPC6 contributes to high glucose-induced apoptosis of podocytes via regulating the RhoA/ROCK^38^ and Wnt/β-catenin pathways^37^. However, there have been no reports regarding the role of TRPC6 and the downstream signaling pathways in the apoptosis of endothelial cells. In this study, we used a TRPC6 gain-of-function approach to explore its role in mediating endothelial apoptosis induced by miR-26a downregulation. We found that with TRPC6 overexpression, miR-26a lost its ability to suppress apoptosis. Although our data strongly support the involvement of TRPC6 repression in the anti-apoptotic effects of miR-26a, we cannot rule out the participation of other genes in this process. In addition to TRPC6, other genes have been previously validated as targets of miR-26a, including phosphatase and tensin homolog (PTEN)^40^, DNA methyltransferase 3B^41^ and enhancer of zeste homolog 2^42^. Particularly, silencing of PTEN has been demonstrated to inhibit high glucose-induced apoptosis in human vascular endothelial cells^43^. Thus, PTEN may also be involved in the anti-apoptotic action of miR-26a in ox-LDL-treated HAECs. In addition, computational analysis predicts that the 3'-untranslated region of transient receptor potential channel 3 (TRPC3) mRNA contains a binding site for miR-26a (http://www.targetscan.org), and a previous study has identified that TRPC3 is involved in regulating cell apoptosis^44^, indicating that TRPC3 may also be a downstream effector of miR-26a in ox-LDL-treated HAECs. Further studies are needed to validate the possible contributions of these genes to the anti-apoptotic effect of miR-26a.

Cytosolic calcium is one of the most well-known intracellular messengers that control cell fate^45^. Disruption of calcium homeostasis, especially an excessive increase in cytosolic calcium can lead to apoptosis^46^. Cytosolic calcium overload activates the mitochondrial apoptotic pathway with the release of cytochrome c and activation of caspase-3^47^. Because TRPC6 is a calcium permeable channel, we speculated that miR-26a may produce an anti-apoptotic effect through inhibiting TRPC6-induced calcium overload. This notion was supported by the facts that forced expression of miR-26a decreased cytosolic calcium and the protein levels of cytochrome c and cleaved caspase-3.

Taken together, our study identified for the first time miR-26a as a novel anti-apoptotic miRNA, which is dysregulated in atherosclerosis and directly targets TRPC6 in vascular endothelial cells. In other words, derepression of TRPC6 due to miR-26a downregulation critically contributes to the endothelial apoptosis in the setting of atherosclerosis. Our findings also unraveled a heretofore unknown pathway involved in endothelial apoptosis: miR-26a-TRPC6-intracellular calcium-cytochrome c-caspase 3. However, it is undeniable that the effect of miR-26a on endothelial cell apoptosis may be regulated by other genes and pathways as well, and the full mechanisms require further investigations. This study provides evidence that strategies aimed at restoring miR-26a expression in endothelial cells may be a promising therapeutic approach for vascular diseases such as atherosclerosis.

## Methods

### Ethics statement

The study was approved by the Animal Care and Use Committee of Harbin Medical University. All experimental procedures were performed in accordance with the Guide for the Care and Use of Laboratory Animals, published by the US National Institutes of Health (NIH Publication No. 85–23, revised 1996).

### Animal models and immunohistology of atherosclerotic lesions

Eight-week-old male ApoE^−/−^ mice were housed under standard animal room conditions (temperature, 21 ± 1°C; humidity, 55–60%). The animals were randomly divided into two groups: the normal-diet group and the HFD group. The animals of the HFD group were subsequently maintained on diet with high fat for 12 weeks to induce atherosclerosis. After twelve weeks, the aortas were carefully excised from the mice. The aortic roots along with the basal portion of the heart were fixed with 4% paraformaldehyde, followed by embedding in OCT compound, and were cut cross-sectionally into 7-μm-thick sections. Atherosclerotic lesions of the aortic root were observed by HE staining. Oil red O staining was performed according to the manufacturer's instructions to show the lipid deposition with an Oil red O staining kit (Nanjing Jiancheng Biology Engineering Institute, Nanjing, Jiangsu, China). In addition, immunostaining was performed on the aortic root for expression of TRPC6 (Abcam, Cambridge, MA, USA). VE-cadherin (Abcam, Cambridge, MA, USA) was used as an endothelial marker. Images were captured using a confocal laser scanning microscope. Fluorescence intensity of TRPC6 was measured using Image-Pro Plus software (Media Cybernetics, Bethesda, MD, USA). The data were calculated from 5 mice for each group. For each mouse, 5 cells were randomly selected for quantification.

### Aorta intima RNA isolation

Aorta intimal RNA was isolated as previously described^11^. Briefly, mouse aortas were exposed, and the surrounding tissues were removed carefully. After perfusion with saline, the aorta was removed and transferred to a dish containing ice-cold PBS. To obtain samples from predilection sites and nonpredilection sites, the aorta was cut into two parts: the aortic arch (predilection sites) and the thoracoabdominal aorta (nonpredilection sites). The preparation was quickly flushed with TRIzol reagent (Invitrogen, Carlsbad, CA, USA) using an insulin syringe, and the eluate was collected in a 1.5-ml tube and prepared for RNA extraction. The aorta leftover (media + adventitia) was stored at −80°C until RNA extraction.

### Cell culture and transfection

HAECs were obtained from ScienCell Research Laboratories (Carlsbad, CA, USA) and cultured in Endothelial Cell Medium supplemented with endothelial cell growth factors, 5% FBS and 1% penicillin/streptomycin. The cells were maintained at 37°C with 5% CO_2_ and 95% air. HAECs were transiently transfected with miR-26a mimics, miR-26a inhibitors or negative controls (RiboBio Co., Ltd., Guangzhou, Guangdong, China), using Lipofectamine 2000 reagent (Invitrogen, CA, Carlsbad, USA) according to the manufacturer's instructions. TRPC6-expressing plasmid was transfected into HAECs. Briefly, cells were trypsinized and seeded for 24 h before transfection. The transfection mixture was dissolved in Opti-MEM serum-free medium and added to the cells. After 24 h of transfection, the medium was replaced by fresh medium with or without ox-LDL. After drug treatment, the cells were used for immunofluorescent staining or protein/RNA extraction.

### Luciferase reporter assay

The luciferase reporter assay was performed as previously described^48^,^49^. Briefly, luciferase reporters containing wild-type or mutated 3'UTR of TRPC6 were constructed using psi-CHECK2 vectors (Promega, Madison, WI, USA). Then, 293T cells were seeded in a 24-well plate and co-transfected with 0.5 μg plasmid and miR-26a mimics or miR-26a inhibitors or negative controls using Lipofectamine 2000 reagent. Renilla luciferase was used as an internal control. Forty-eight hours after transfection, the cells were collected, and firefly and Renilla luciferase activities were evaluated using Dual-Luciferase Reporter Assay System (Promega, Madison, WI, USA).

### Cell viability assay

MTT assay was performed to evaluate cell viability of HAECs as previously described^50^. Briefly, cells were seeded in 96-well plates followed by miRNA or drug treatments for 24 h. A total of 20 μl of MTT solution was added to each well, and the cells were incubated for 4 h. A total of 150 μl of DMSO was added to dissolve the formazan crystals. Absorbance at 570 nm was measured using a plate reader.

### TUNEL staining

TUNEL staining was used to detect DNA fragmentation of individual cells as described in our previous work^51^ using a TUNEL fluorescence FITC kit (Roche, Indianapolis, IN, USA). For tissues, after TUNEL staining, the aorta sections were immersed into DAPI solution to stain nuclei. For cells, HAECs grown on coverslips were fixed with 4% paraformaldehyde followed by permeabilization with 0.1% Triton X-100. Then, cells were incubated with TUNEL reaction mixture at 37°C for 1 h. The stained tissues and cells were examined under a confocal laser scanning microscope (FV300, Olympus, Japan).

### Cytosolic calcium evaluation

Cells seeded on the coverslips were washed thrice with a buffer containing calcium ions (136 mM NaCl, 5.4 mM KCl, 10 mM HEPES, 0.33 mM NaH_2_PO_4_.2H_2_O, 1 mM MgCl_2_.6H_2_O, 1 mM glucose and 1.8 mM CaCl_2_, pH, 7.4). Then, the cells were incubated with 5 μmol/L Fluo-3/AM (Molecular Probe, Eugene, OR, USA) for 35–45 min at 37°C. After rinsing with the calcium buffer, the cells were visualized under a confocal laser scanning microscope (FV300, Olympus, Japan, 495 nm for excitation and 525 nm for emission).

### Real-time RT-PCR

Total RNA was harvested from tissues and cells using TRIzol reagent (Invitrogen, CA, USA) according to the manufacturer's protocols. The extracted RNA was reverse transcribed into cDNA using High-Capacity cDNA Reverse Transcription Kit (Applied Biosystems, Foster City, CA, USA). The first-strand cDNA was used for real-time PCR to quantify mRNA expressions of CD31 and smMHC with GADPH as an internal control. miR-26a expression was detected with U6 as an internal control. The RT primer and forward and reverse primer pairs for miR-26a were designed by RiboBio Co., Ltd. (Guangzhou, Guangdong, China). CD31, smMHC and miR-26a levels were presented as values of 2^−ΔΔCt^. The sequences of primers are as follows:

CD31 forward, 5'- ACGCTGGTGCTCTATGCAAG-3' and reverse, 5'-TCAGTTGCTGCCCATTCATCA-3'; smMHC forward, 5'- AAGCTGCGGCTAGAGGTCA-3' and reverse, 5'- CCCTCCCTTTGATGGCTGAG-3'; and GAPDH forward, 5'-AAGAAGGTGGTGAAGCAGGC-3' and reverse, 5'-TCCACCACCCAGTTGCTGTA-3'.

### Western blotting

Western blot analysis was performed as previously described^52^,^53^. The cells were lysed with RIPA buffer containing protease and phosphatase inhibitors. After centrifugation, the supernatant was collected and quantified. The proteins were then separated by SDS-PAGE and transferred to nitrocellulose membranes. After blocking with 5% non-fat milk, the membranes were probed with anti-TRPC6 (Abcam, Cambridge, MA, USA), anti-cytochrome c (Santa Cruz Biotechnology, Dallas, TX, USA), anti-caspase-3 (Cell Signaling Technology, Danvers, MA, USA), and anti-cleaved caspase-3 (Cell Signaling Technology, Danvers, MA, USA), followed by incubation with a florescence-labeled secondary antibody. Western blot bands were scanned using the Odyssey Imaging System (LI-COR, Lincoln, NE, USA). GAPDH (Zhongshanjinqiao, Inc., Beijing, China) was used as internal control.

### Immunofluorescence

Immunofluorescent staining was performed as previously described^54^,^55^. After appropriate treatment, HAECs were fixed with 4% paraformaldehyde followed by permeabilization with 0.4% Triton X-100. Cells were blocked with goat serum and incubated with TRPC6 antibody. Then, the cells were probed with florescence-labeled secondary antibody and observed under a confocal laser scanning microscope (FV300, Olympus, Japan).

### Statistical analyses

The data in this study are shown as the mean ± S.E.M. Differences among groups were analyzed using one-away ANOVA accompanied with Turkey multiple-comparisons test (GraphPad Prism version 5.0). Two-tailed Student's t-test was used for comparison between two groups; p < 0.05 was considered significant.

### Drugs and chemicals

Ox-LDL was obtained from Beijing Xiesheng Bio-Technology Limited (Beijing, China). κ-carrageenan was obtained from Aladdin Industrial Inc. (Shanghai, China).

## Author Contributions

B.F.Y. and Y.Z. designed the study. W.Q., L.Y.Z., X.X.W., N.D., Y.Y.H., X.G.L., N.N.S., D.X., H.Y.Z., Z.G.L., Y.Z., H.Y. and F.G. carried out data acquisition and analysis. Y.Z. and W.Q. wrote the paper. C.Q.X., Z.M.D. and B.F.Y. supervised the study. All authors reviewed the manuscript.

## Supplementary Material

Supplementary Information Supplementary information
