# Supplementary material for: MicroRNA-26a prevents endothelial cell apoptosis by directly targeting TRPC6 in the setting of atherosclerosis
Source: Sci Rep. 2015 Mar 24;5:9401. doi: 10.1038/srep09401 (PMC4371083; doi:10.1038/srep09401)
Supplement: Supplementary Information [file srep09401-s1.pdf]

MicroRNA-26a prevents endothelial cell apoptosis by directly targeting TRPC6 in the setting of atherosclerosis

Yong Zhang[1,2,#], Wei Qin[1,#], Longyin Zhang[1], Xianxian Wu[1], Ning Du[1], Yingying Hu[1], Xiaoguang Li[1], Nannan Shen[1], Dan Xiao[1], Haiying Zhang[1], Zhange Li[1], Yue Zhang[1], Huan Yang[1], Feng Gao[1], Zhimin Du[3], Chaoqian Xu[1,2], Baofeng Yang[1,2,*]

[1]Department of Pharmacology (State-Province Key Laboratories of Biomedicine-Pharmaceutics of China, Key Laboratory of Cardiovascular Research, Ministry of Education), Harbin Medical University, Harbin 150081, China

[2]Institute of Cardiovascular Research, Harbin Medical University, Harbin 150081, China

[3]Institute of Clinical Pharmacy, The Second Affiliated Hospital of Harbin Medical University, Harbin 150081, China

[#]These authors contributed equally to this work.

*Corresponding author: Prof. Baofeng Yang, Department of Pharmacology, Harbin Medical University, 157 Baojian Road, Nangang District, Harbin, Heilongjiang 150081, China. Tel & Fax: +86-451-86671354; E-mail: yangbf@ems.hrbmu.edu.cn

Supplementary Information

Supplementary Methods

In situ detection of mature miR-26a

The aorta was fixed in 4% paraformaldehyde, and embedded in paraffin. Then, the preparations were cut cross-sectionally into 5-μm thick sections, which were hybridized with double digoxigenin (DIG)-labeled probes for miR-26a (Exiqon, Woburn, MA, USA). The sections were then incubated with alkaline phosphatase labeled anti-DIG antibody (anti-DIG-AP) (Roche, Indianapolis, IN, USA), followed by BCIP/NBT developing solution and visualized under microscopy.

Mouse model with partial ligation of the carotid artery

Low shear stress was generated by partial ligation of the carotid artery in $ApoE^{-/-}$ mice as described previously.[1] In brief, $ApoE^{-/-}$ mice were anesthetized with pentobarbital sodium (6 mg kg^{-1}, i.p.) as assessed by interdigital reflex. Then, the left external and internal carotid, and occipital artery, but not the superior thyroid artery, were ligated with 6-0 silk sutures. Three days after partial ligation, the carotid artery of the mice was removed and the intima was collected quickly by flushing with TRIzol reagent (Invitrogen, Carlsbad, CA, USA) using an insulin syringe. All experimental procedures were approved by the Animal Care and Use Committee of Harbin Medical University and complied with the Guide for the Care and Use of Laboratory Animals, published by the US National Institutes of Health (NIH Publication No. 85–23, revised 1996).

Supplementary Figures

Supplementary Figure S1

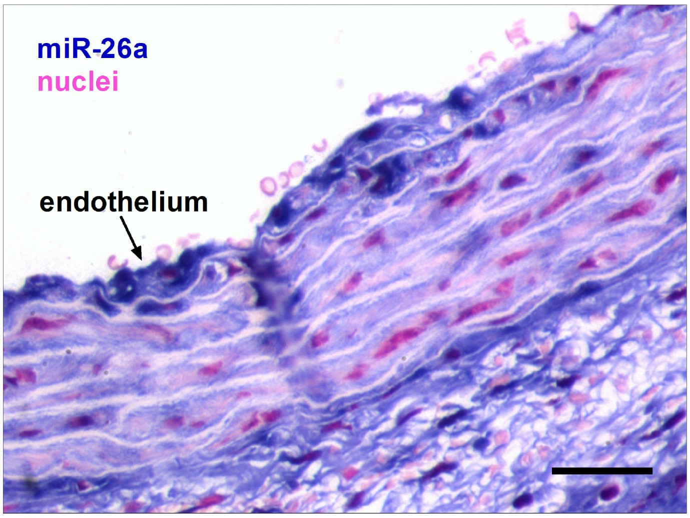

Aorta of ApoE$^{-/-}$ mouse

Legend S1. MiR-26a is expressed in the endothelium in vivo. Paraformaldehyde fixed ApoE$^{-/-}$ mouse aorta was hybridized with probes for miR-26a (blue). The nuclei were stained by nuclear fast red. Scale bar indicates 200 μm.

Supplementary Figure S2

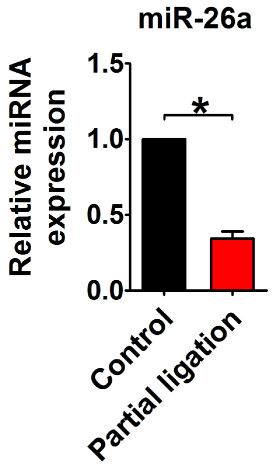

Legend S2. Repression of miR-26a level by low shear stress. miR-26a expression in the intima after partial ligation of the left carotid artery. Carotid artery without partial ligation was used as control. n=5 mice in each group. The data are presented as the mean ± S.E.M., * $p<0.05$.

Supplementary Figure S3

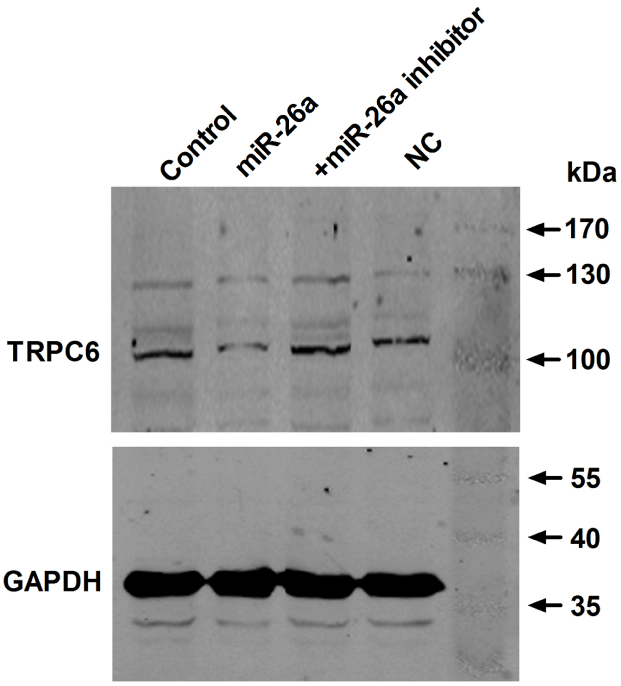

Legend S3. Full-length blots of Figure 5(a) in the main text. Effect of miR-26a on the protein level of TRPC6. miR-26a indicates miR-26a mimics. +miR-26a inhibitor indicates co-application of miR-26a mimics and miR-26a inhibitor. NC indicates miRNA negative control.

Supplementary Figure S4

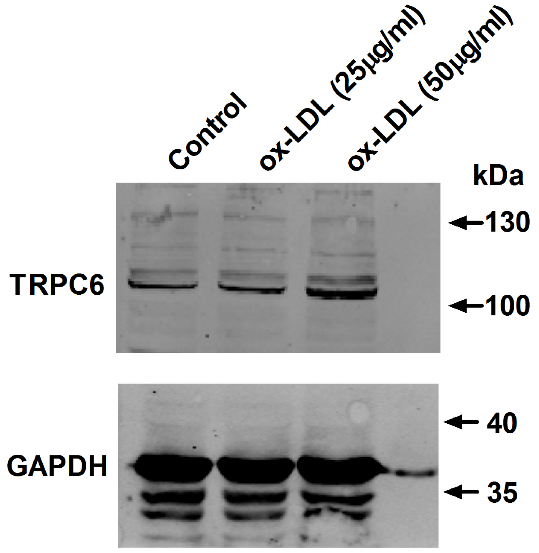

Legend S4. Full-length blots of Figure 5(b) in the main text. Effect of ox-LDL on the protein level of TRPC6.

Supplementary Figure S5

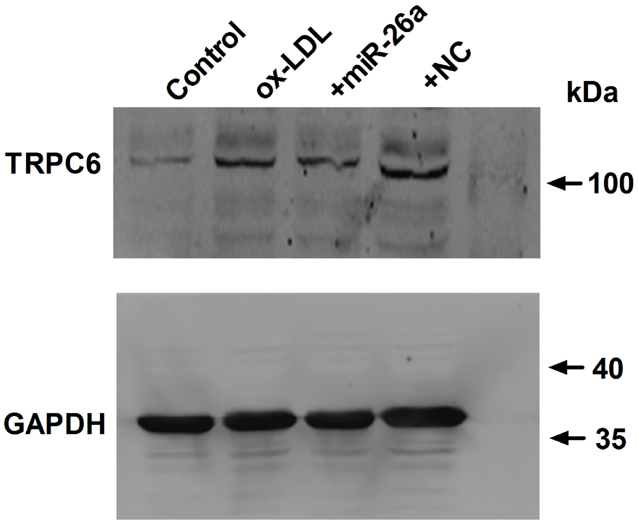

Legend S5. Full-length blots of Figure 5(c) in the main text. The ability of miR-26a to repress ox-LDL-induced TRPC6 expression. ox-LDL indicates ox-LDL (50 μg/ml). +miR-26a indicates co-application of miR-26a mimics and ox-LDL (50 μg/ml). +NC indicates co-application of miRNA negative control and ox-LDL (50 μg/ml).

Supplementary Figure S6

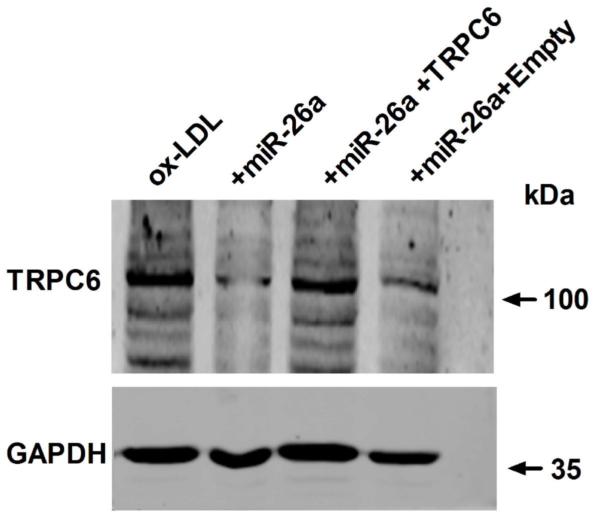

Legend S6. Full-length blots of Figure 6(a) in the main text. Protein levels of TRPC6 detected by Western blot. ox-LDL indicates ox-LDL (50 μg/ml). +miR-26a indicates co-application of miR-26a mimics and ox-LDL (50 μg/ml). +miR-26a+TRPC6 indicates co-application of miR-26a mimics, ox-LDL (50 μg/ml) and TRPC6 plasmids. +miR-26a+Empty indicates co-application of miR-26a mimics, ox-LDL (50 μg/ml) and empty plasmids.

Supplementary Figure S7

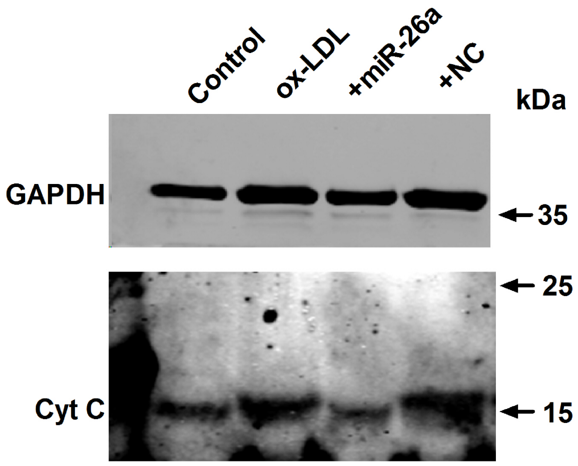

Legend S7. Full-length blots of Figure 7(b) in the main text. Protein levels of cytochrome c (Cyt C) detected by Western blotting. ox-LDL indicates ox-LDL (50 μg/ml). +miR-26a indicates co-application of miR-26a mimics and ox-LDL (50 μg/ml). +NC indicates co-application of miRNA negative control and ox-LDL (50 μg/ml).

Supplementary Figure S8

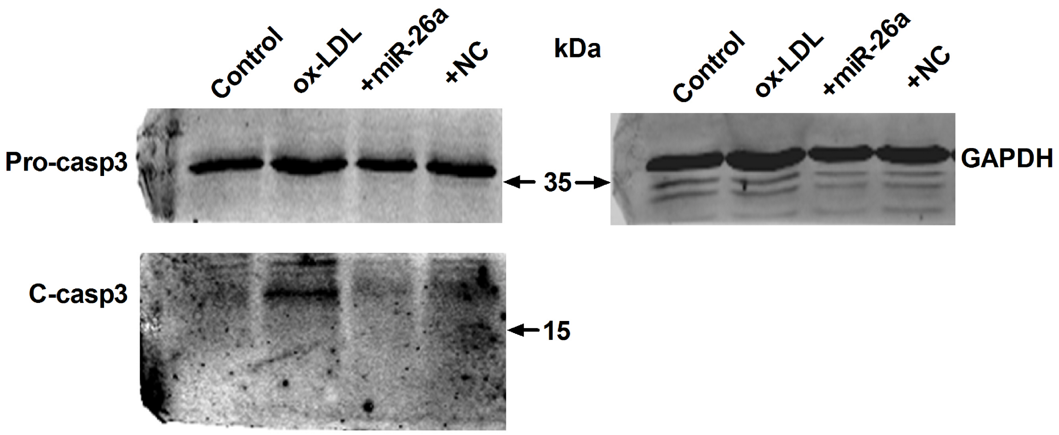

Legend S8. Full-length blots of Figure 7(c) in the main text. Western blot analysis of proform and active cleaved form of caspase3. Pro-casp3 indicates proform of caspase-3 and C-casp3 indicates cleaved form of caspase-3. ox-LDL indicates ox-LDL (50 µg/ml). +miR-26a indicates co-application of miR-26a mimics and ox-LDL (50 µg/ml). +NC indicates co-application of miRNA negative control and ox-LDL (50 µg/ml).

References

1 Schober, A. *et al.* MicroRNA-126-5p promotes endothelial proliferation and limits atherosclerosis by suppressing Dlk1. *Nat. Med.* **20,** 368-376 (2014).
